# Albiflorin inhibits inflammation to improve liver fibrosis by targeting the CXCL12/CXCR4 axis in mice

**DOI:** 10.3389/fphar.2025.1577201

**Published:** 2025-04-30

**Authors:** Lingjie Meng, Huijing Lv, Anli Liu, Qian Cao, Xinyi Du, Chengjin Li, Qinggui Li, Qingqing Luo, Yi Xiao

**Affiliations:** ^1^ Institute of life sciences, Zunyi Medical University, Zunyi, Guizhou, China; ^2^ College of Basic Medicine, Zunyi Medical University, Zunyi, Guizhou, China

**Keywords:** albiflorin, CXCL12/CXCR4 axis, liver fibrosis, inflammation, combination therapeutic

## Abstract

Liver fibrosis is a common pathological feature of chronic hepatic injury that currently lacks effective therapeutic interventions. Albiflorin (ALB), a pinane-type monoterpene derived from *Paeonia lactiflora* Pall, has notable anti-inflammatory and hepatoprotective effects. However, the potential role of ALB against liver fibrosis is largely unknown. In this study, we discovered that ALB significantly inhibited CCl_4_-induced liver fibrosis in mice. This was evidenced by improvements in liver and kidney function indexes, fibrosis indicators, and histopathological findings. *In vitro* studies also showed that ALB inhibited TGF-β1-induced LX-2 cell activation and reduced the expression of α-SMA and collagen I. Additionally, we found that ALB mitigates inflammation and ameliorates liver fibrosis by targeting the CXCL12/CXCR4 axis, as confirmed using the CXCR4 inhibitor AMD3100 in CCl_4_-treated mice. Notably, combining ALB with metformin (MET) enhanced the inhibition of liver fibrosis progression. These findings highlight that ALB exerts anti-liver fibrosis effects by targeting the CXCL12/CXCR4 axis, underscoring its potential as a standalone treatment or as an adjuvant therapy.

## Introduction

Liver fibrosis commonly arises from persistent liver damage, including conditions such as viral hepatitis, alcoholic associated liver disease, and metabolic syndrome-associated steatohepatitis (Hammerich and Tacke, 2023). Recurrent insults precipitate a cascade of inflammatory responses, culminating in the advancement of fibrosis or its progression to cirrhosis in the absence of effective intervention (Tung et al., 2024). The activation of hepatic stellate cells (HSCs) constitutes a pivotal mechanism in the pathogenesis of liver fibrosis (Yang et al., 2024). When activated, HSCs transform into myofibroblast-like cells, marked by α-smooth muscle actin (α-SMA) expression. This transformation is instrumental in the excessive deposition of extracellular matrix (ECM) components, including collagen and proteoglycans, thereby facilitating the progression of liver fibrosis (Jiang et al., 2024). Thus, inhibition of HSCs activation and/or pro-fibrotic cytokines has been considered as one strategy for liver fibrosis treatment.

CXCL12, a small molecular cytokine involved in inflammatory responses and a member of the chemokine family, is recognized as the unique ligand that binds to and activates the CXCR4 receptor. Upon binding of CXCL12 to CXCR4, a conformational change initiates a series of intracellular signal transduction cascades (Janssens et al., 2018; Liu et al., 2020; Wu et al., 2023). Notably, activation of the CXCL12/CXCR4 signaling axis has been shown to exacerbate hepatic inflammation and fibrosis (Aguilar-Bravo et al., 2023; Zhang et al., 2023). Moreover, activated HSCs express functional CXCR4 receptors; upon binding with CXCL12, this interaction induces a pro-fibrotic phenotype, culminating in augmented expression of *α*-SMA and Collagen I, key markers of fibrosis progression (Hong et al., 2009).

Albiflorin (ALB) is a water-soluble monoterpene glycoside primarily found in the root of *Paeonia lactiflora*, also known as ‘Chishao'. Research has shown that ALB reduces inflammation and oxidative stress associated with colitis and exhibits anti-neuroinflammatory effects (Zhao et al., 2021). Emerging evidence highlights ALB’s hepatoprotective properties, with mechanistic studies demonstrating its capacity to inhibit P38 MAPK/NF-κB signaling in pancreatitis-associated hepatic injuryand mitigate thioacetamide-induced fibrogenesis through hepatotoxicant detoxification (Li H. et al., 2024; Ou et al., 2024; Zhang H. et al., 2022; Song et al., 2024). Nonetheless, due to the limited research on ALB’s effectiveness in treating liver fibrosis, its potential protective effects against CCl_4_-induced liver fibrosis have not been thoroughly investigated.

In this study, we explored the role of ALB in anti-liver fibrosis. Interestingly, we discovered that ALB not only inhibits inflammation to improve liver fibrosis by targeting the CXCL12/CXCR4 axis, but also synergizes with MET to inhibit the progression of liver fibrosis. Our study highlights ALB’s potential both alone and as an adjuvant therapy.

## Materials and methods

### Drugs and reagents

Paeoniflorin (#T2230) were provided by TargetMol (Shanghai, China). AMD3100 (#1115-70-4) and Metformin hydrochloride (#155148-31-5) were purchased from Aladdin Biochemical Technology Co. (Shanghai, China). Antibodies against α-Smooth Muscle Actin (#ET1607-53), p38 (#ET1702-65), p-p38 (#HA722150), STAT3 (#ET1607-38), CXCL12 (#ER1902-35), JAK1 (#ET1705-84) and GAPDH (#ET1601-4) were obtained from Huabio (Hangzhou, China). Antibodies against Collagen I (#ab270993), CXCL12 (#ab155090) were obtained from abcam. (Cambridge, UK). The antibody against CXCR4 (#60042-1-Ig) was purchased from Proteintech (Wuhan, China). The antibody against p-STAT3 (#T156566) was purchased from Abmart (Shanghai, China). The antibody against p-JAK1 (#T156566) was purchased from abcepta (Jiangsu, China). The antibody against Collagen I (#72026T) was purchased from Cell Signaling Technology (USA).

### Animals and reagents

Male C57BL/6J mice (6–8 weeks old) were housed at the Animal Center of Zunyi Medical University with free access to food and water. All experiments were conducted following the ethical guidelines approved by the Committee on Animal Experimentation of Zunyi Medical University (Approval Number: ZMU21-2403-034). Carbon tetrachloride (CCl_4_) and corn oil were supplied by Macklin (Shanghai, China).

### Animal model of liver fibrosis and experimental design

The CCl_4_-induced liver fibrosis model was established according to our previously published research(Meng et al., 2024). Briefly, Mice in the model group received intraperitoneal injections of a 20% CCl_4_ and corn oil mixture every 2 days. The initial dose was 2 mL/kg, which was increased to 3 mL/kg during the first and second weeks, and further elevated to 4 mL/kg during the third and fourth weeks.

To evaluate the effect of ALB on liver fibrosis in CCl_4_-treated mice, 24 mice were randomly divided into four groups of six mice each: the control group, the CCl_4_ group, the ALB group (100 mg/kg), and the paeoniflorin group (50 mg/kg). Notably, We previously demonstrated that paeoniflorin (alone or combined with metformin) inhibits liver fibrosis in mice; thus, it was employed as a positive control in our experiments (Meng et al., 2024). The mice received daily intraperitoneal injections of the respective drugs or an equivalent volume of saline for 14 consecutive days. Mice were sacrificed 24 h after the last dose, and liver tissues were collected for further analyses.

To explore the potential role of the CXCL12/CXCR4 axis in ALB protection against liver fibrosis, a total of 30 mice were randomly divided into five groups of 6 mice each, including the control group, the CCl_4_ group, the ALB group, the AMD3100 group (a CXCR4 inhibitor), and theALB plus AMD3100 group. Mice received intraperitoneal injections of ALB (100 mg/kg), AMD3100 (5 mg/kg), or the same volume of saline solution for 14 consecutive days. Mice were sacrificed 24 h after the last dose, and liver tissues were collected for further analyses.

To study the effects of the combined use of ALB and MET on liver fibrosis in mice,a total of 30 mice were randomly divided into five groups of 6 mice each, including the control group, the CCl_4_ group, the ALB group, MET group, and the ALB plus MET group. Mice received intraperitoneal injections of ALB (50 mg/kg), MET (100 mg/kg), or the same volume of saline solution for 14 consecutive days. Mice were sacrificed 24 h after the last dose, and liver tissues were collected for further analyses.

### Cell culture and treatments

The human hepatic stellate cell line LX-2 was obtained from the BeNa Culture Collection in Henan, China. To establish an *in vitro* model for evaluating the effect of ALB on the activation of hepatic stellate cells, LX-2 cells were seeded in 6-well plates at a density of 1.5 × 10^5^ cells per well in RPMI-1640 mediumwith 10% FBS and cultured at 37 °C under 5% CO_2_. After a 24-hour incubation period, both the model group and the ALB group were exposed to TGF-β1 (20 ng/mL) for an additional 24 h. The control group received only the same volume of RPMI-1640 medium without TGF-β1. Subsequently, the ALB group was treated with a specific concentration of ALB solution for 24 h. Following these treatments, the cells were harvested for further experimentation.

### Western blot analysis

The RIPA lysis buffer was used to extract total proteins from liver tissue and LX-2 cells. After BCA protein quantification, SDS-PAGE electrophoresis, membrane transfer, incubation with primary antibodies, incubation with secondary antibodies, and visualization, the expression levels of relevant proteins in the liver tissue and LX-2 cells were detected.

### Quantitative real-time PCR

Total RNA was extracted from liver tissue and LX-2 cells using the Vazyme Trizol Total RNA Extraction Kit (Nanjing, China). After determining the RNA concentration, the RNA was reverse transcribed into cDNA using the HiScript^®^ II Q RT SuperMix for qPCR reverse transcription kit (Vazyme, Nanjing, China) according to the manufacturer’s instructions. The real-time qPCR reaction system was prepared following the guidelines for the ChamQ Universal SYBR qPCR Master Mix (Vazyme, Nanjing, China), and the real-time qPCR reactions were performed according to the recommended protocol. Using GAPDH as the internal reference, the mRNA expression levels were calculated using the 2^−△△Ct^ method. The specific primer sequence is shown in Supplementary Table S1.

### Histopathological analysis

After euthanizing the mice, liver tissues were collected. A portion of each liver tissue sample was immediately immersed in formalin solution for fixation over 24 h. Subsequently, the specimens were dehydrated and embedded in paraffin. Tissue sections were cut to a thickness of 4.5 μm. After dewaxing and rehydration, the sections were stained with hematoxylin-eosin (H&E) to observe histopathological damage and inflammatory response. Sirius Red staining and Masson’s trichrome staining were used to assess the degree of fibrosis. The stained sections were examined under a light microscope (Olympus, Tokyo, Japan), and quantitative analysis of Sirius Red and Masson’s trichrome staining was performed using ImageJ software.

### RNA-sequencing analysis

Total RNA was isolated using TRIzol reagent. The concentration of the RNA was measured using the Nanodrop 2000 (Thermo Fisher Scientific, USA), andthe integrity of the RNA was subsequently evaluated using an Agilent 2100 Bioanalyzer (Agilent Technologies, USA). RNA library sequencing was then performed on the Illumina NovaSeq X Plus platform (San Diego, USA). Differential expression analysis between samples was conducted using DESeq2 software. Genes with a fold change greater than 2 and a *P*-value less than 0.01 were defined as differentially expressed genes. GO and KEGG enrichment analyses were performed on these genes. The raw sequencing data have been submitted to the NCBI Gene Expression Omnibus (GEO) under the accession number GSE282681.

### Statistical analysis

The data are presented as the mean ± standard deviation (SD) from a minimum of three independent experiments. Significant differences among means were evaluated using one-way ANOVA followed by the Tukey post-hoc test in GraphPad Prism 7.0 software. *P*-values <0.05 were considered statistically significant.

## Results

### ALB ameliorated CCl_4_-induced liver fibrosis in mice

**FIGURE 1:**
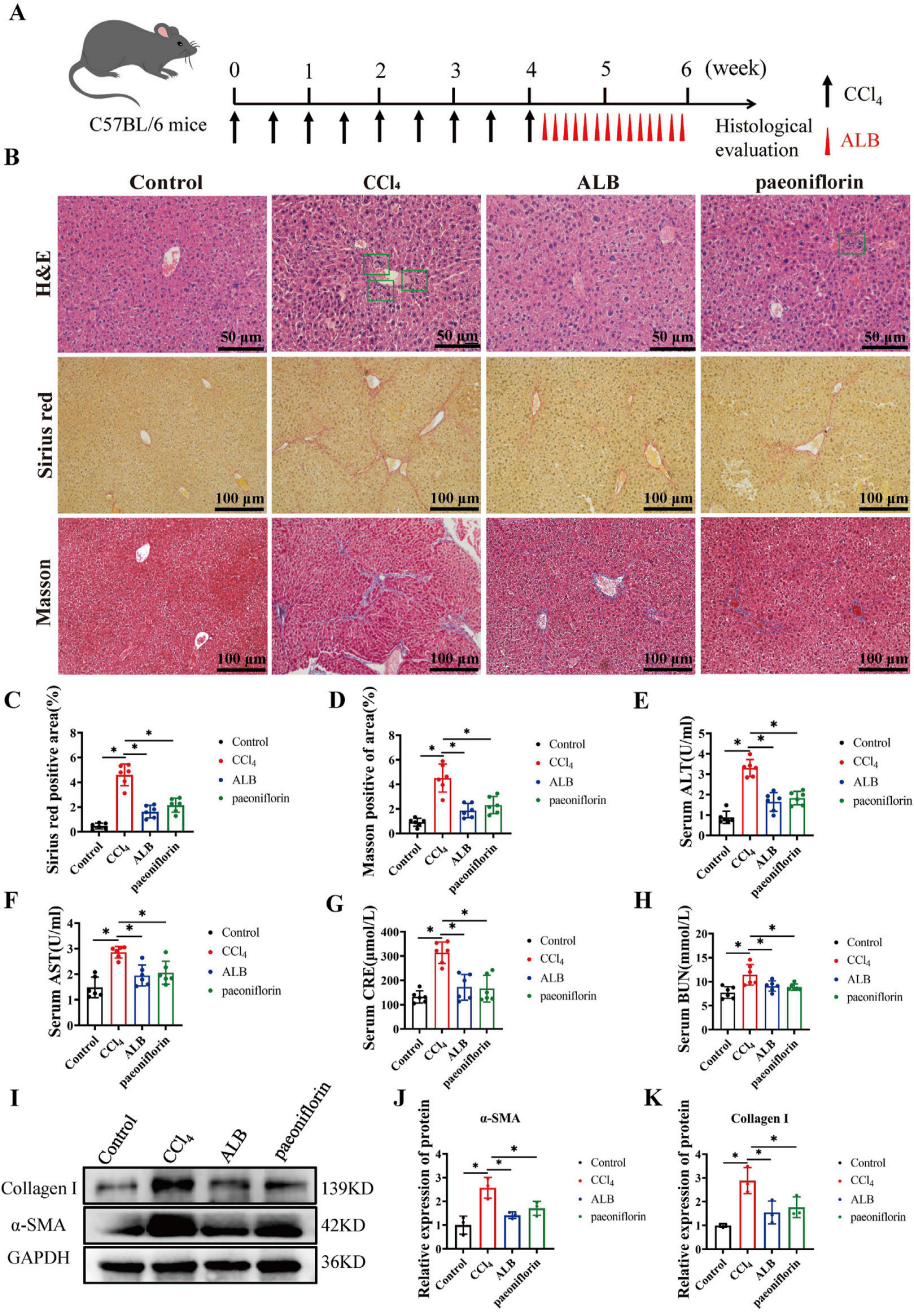
ALB ameliorated CCl_4_-induced liver fibrosis in mice. **(A)** CCl_4_-induced mice liver fibrosis model and treatment regimens. **(B)** H&E staining (bar = 50 μm), Sirius Red staining (bar = 100 μm), and Masson’s trichrome staining (bar = 100 μm) of various experimental groups. **(C)** Quantitative analysis of collagen fibers in Sirius Red-stained sections (n = 6; *P < 0.05). **(D)** Quantitative analysis of collagen fibers in Masson’s trichrome-stained sections (n = 6; *P < 0.05). **(E)** Serum levels of ALT, **(F)** Serum levels of AST, **(G)** Serum levels of CRE, **(H)** Serum levels of BUN. **(I)** Protein expression of α-SMA and Collagen **(I)**. **(J)** Relative protein expression of α-SMA (n = 3; *P < 0.05). **(K)** Relative protein expression of Collagen I (n = 3; *P < 0.05).

Figure 1A illustrates the liver fibrosis model and treatment regimen. To evaluate the impact of ALB on CCl_4_-induced liver fibrosis, we performed histopathological examinations. H&E staining revealed that the CCl_4_ group had thicker fibrotic septa, hepatocyte necrosis, and increased inflammatory cell infiltration compared to controls, which ALB (100 mg/kg) treatment improved. Furthermore, Masson’s trichrome and Sirius red staining showed increased collagen deposition and abnormal collagen fiber proliferation in the CCl_4_ group, both of which were significantly reduced by ALB treatment (Figures 1B–D). Additionally, serum biochemical analyses indicated no hepatorenal toxicity from ALB (Figures 1E–H). Meanwhile, Western blot analysis showed elevated α-SMA and COL1A1 in the CCl_4_ group, with reductions in the ALB-treated and positive control groups (Figures 1I–K). These findings suggest that ALB ameliorated CCl_4_-induced liver fibrosis in mice.

### ALB inhibited TGF-β1-induced LX-2 cell activation via the CXCL12/CXCR4 axis

**FIGURE 2:**
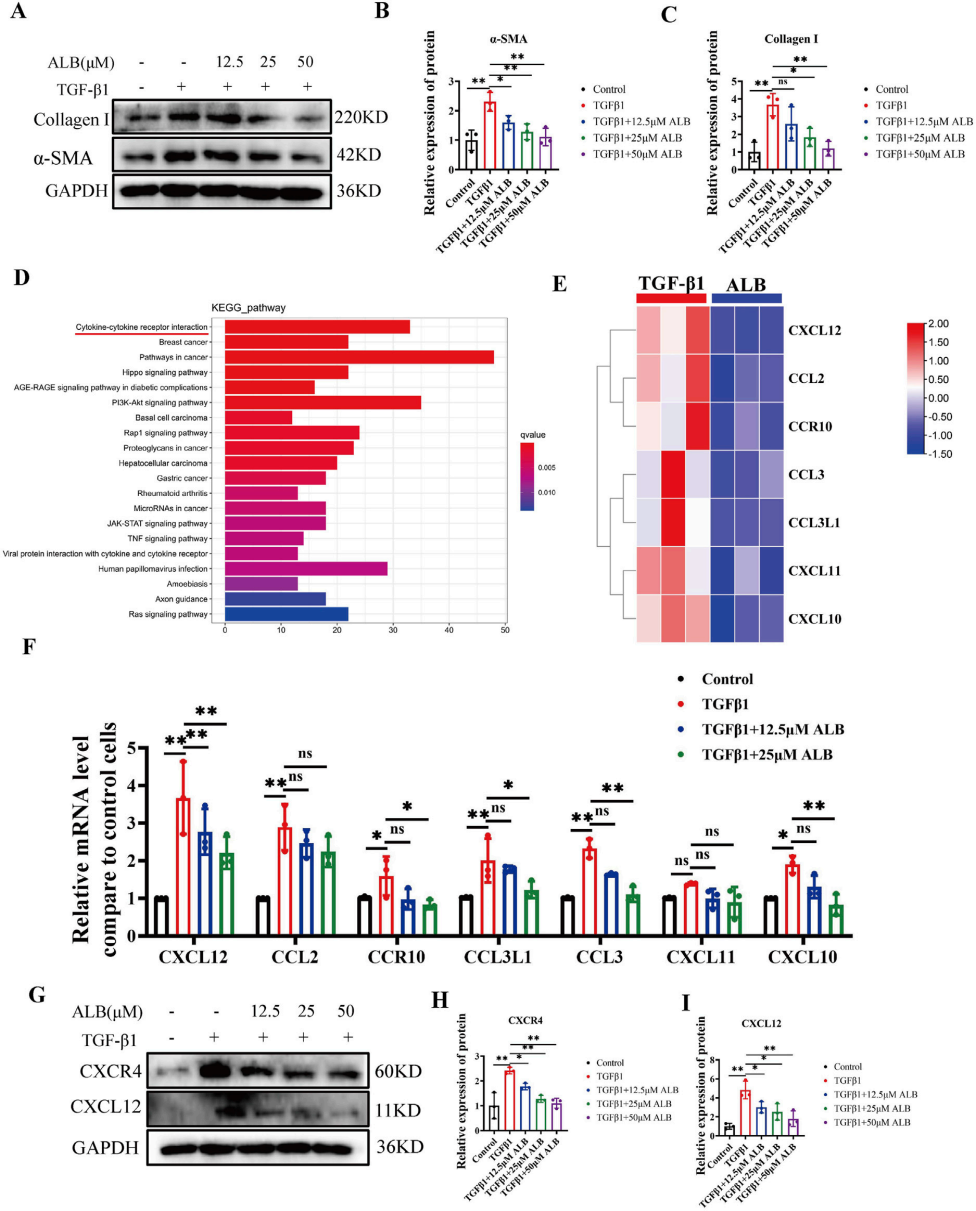
ALB inhibited TGF-β1-induced LX-2 cell activation via the CXCL12/CXCR4 axis. **(A)** Protein expression of α-SMA and Collagen I in TGF-β1-induced LX-2 cells and treated with different concentrations of ALB. **(B)** Relative protein expression of α-SMA (n = 3; *P < 0.05, **P < 0.01). **(C)** Relative protein expression of Collagen I (n = 3; ns, *P* > 0.05, *P < 0.05, **P < 0.01). **(D)** KEGG enrichment analysis. **(E)** Heatmap analysis of gene expression profiles related to chemokine-chemokine receptor interactions in the RNA-seq dataset. **(F)** mRNA expression of genes related to chemokine-chemokine receptor interactions (n = 3; ns, *P* > 0.05, *P < 0.05, **P < 0.01). **(G)** Protein expression of CXCR4 and CXCL12. **(H)** Relative protein expression of CXCR4 (n = 3; *P < 0.05, **P < 0.01). **(I)** Relative protein expression of CXCL12 (n = 3; *P < 0.05, **P < 0.01).

Activated HSCs are the primary cellular source of ECM, with TGF-β1 being the predominant cytokine in activated HSCs (Bai et al., 2023). To investigate the impact of ALB on HSCs activation, we determined the levels of *α*-SMA and Collagen I in TGF-β1activatedLX-2 cells. Figures 2A–C illustrated that activated HSCs exhibited significantly elevated levels of α-SMA and Collagen I, which were notably reduced in a dose-dependent manner following ALB treatment. To further elucidate the mechanism of ALB, we analyzed the RNA-seq data from TGF-β1induced LX-2 cell treated with and without ALB (Supplementary Figures S1A,B). According to the KEGG pathway analysis, the cytokine-cytokine receptor interaction pathway is among the top 3 pathways (Figure 2D). Our results showed that TGF-β1 significantly increased mRNA expression levels of seven chemokine genes in LX-2 cells. Following ALB treatment, this upregulation was reduced, particularly for CXCL12, which showed the most significant decrease in expression (Figures 2E,F). Furthermore, the protein expression levels of the CXCL12 and CXCR4 also exhibited a downward trend in the ALB-treated group (Figures 2G–I). These results suggested that ALB inhibited TGF-β1induced LX-2 cell activation via the CXCL12/CXCR4 axis.

### ALB inhibited the activation of CXCL12/CXCR4 axis and the expression of inflammatory factors in CCl_4_-induced mice

**FIGURE 3:**
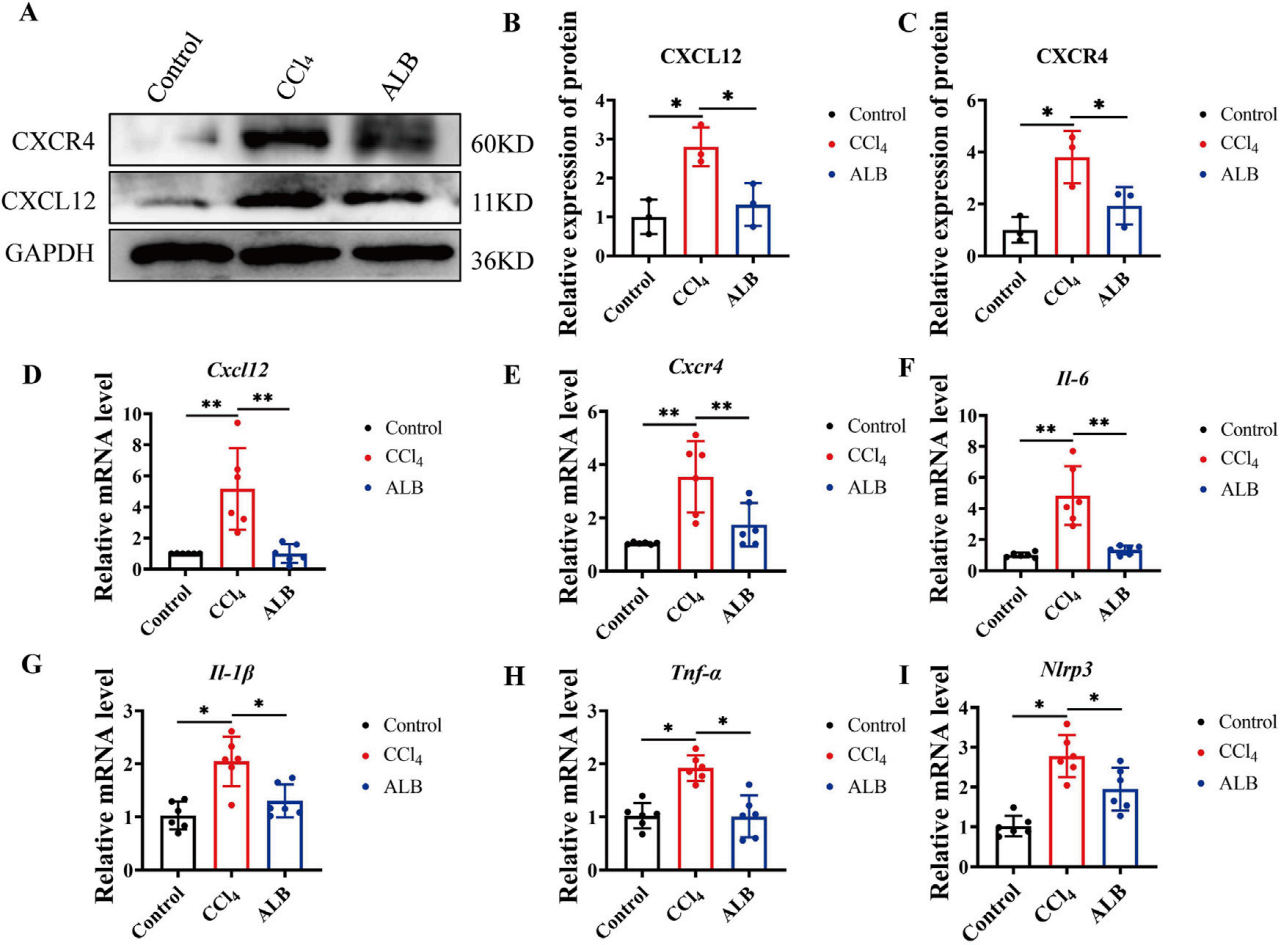
ALB inhibited the activation of CXCL12/CXCR4 axis and the expression of inflammatory factors in CCl_4_-induced Mice. **(A)** Protein expression of CXCL12 and CXCR4. **(B)** Relative protein expression of CXCR4 (n = 3; *P < 0.05). **(C)** Relative protein expression of CXCL12 (n = 3; *P < 0.05). **(D)** Relative mRNA expression of CXCL12 (n = 6; *P < 0.05, **P < 0.01). **(E)** Relative mRNA expression of CXCR4 (n = 6; *P < 0.05, **P < 0.01). **(F–I)** Relative mRNA expression of inflammatory cytokines (n = 6; *P < 0.05).

Based on the aforementioned experimental results, we speculated that ALB can inhibit the activation of the CXCL12/CXCR4 axis in CCl_4_-induced mice. The findings from Western blot (Figures 3A–C) and RT-qPCR (Figures 3D,E) analysis support our hypothesis. (The materials used in the experiment correspond to those illustrated in Figure 1). In the model group, the protein and mRNA expression levels of CXCL12 and CXCR4 were elevated. Conversely, mice treated with ALB exhibited a significant downregulation of these expression levels. Additionally, analysis of inflammationrelated factors (IL-6, IL-1β, TNF-α, and NLRP3) revealed significant downregulation following ALB treatment (Figures 3F–I). These findings suggested that ALB provides hepatoprotection against CCl_4_-induced liver fibrosis by suppressing the CXCL12/CXCR4 axis activation and mitigating inflammation.

### CXCR4 inhibitor abolished the hepaprotective effect of ALB

**FIGURE 4:**
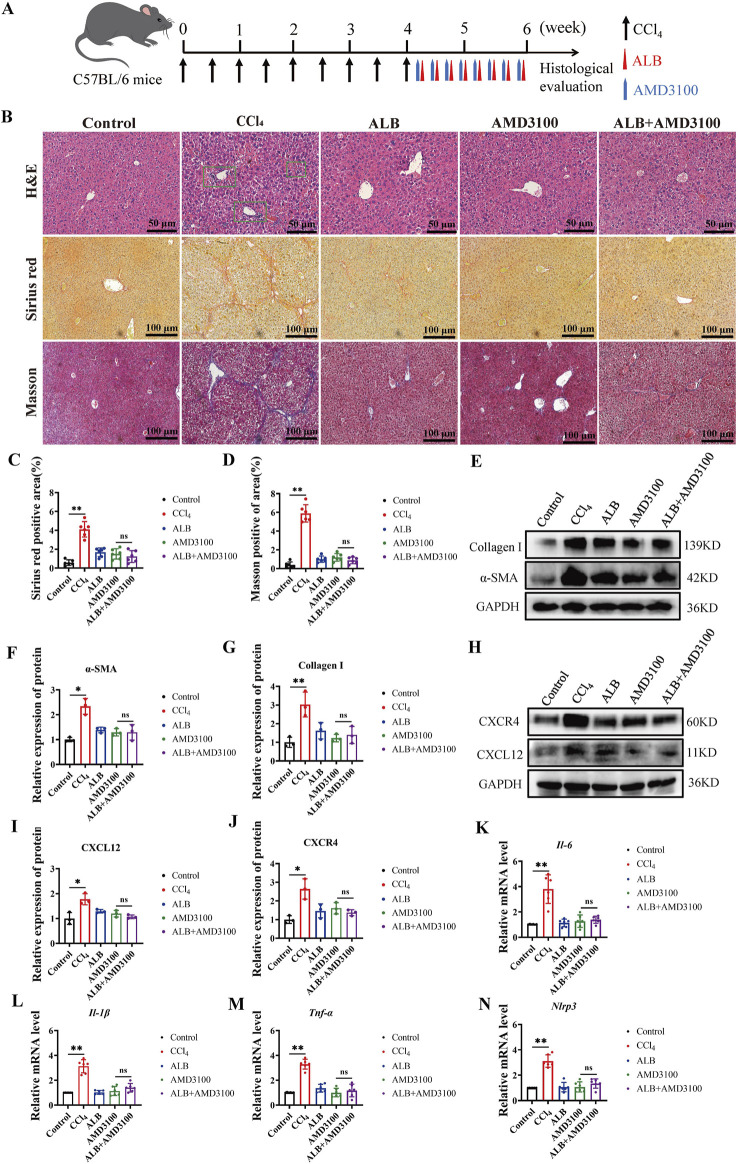
CXCR4 inhibitor abolished the hepaprotective effect of ALB CCl_4_-induced Mice. **(A)** Dosing methods for the co-treatment of AMD3100 and ALB in CCl_4_-induced mouse. **(B)** H&E staining (bar = 50 μm), Sirius Red staining (bar = 100 μm), and Masson’s trichrome staining (bar = 100 μm) of various experimental groups. **(C)** Quantitative analysis of collagen fibers in Sirius Red-stained sections (n = 6; ns, *P* > 0.05, **P < 0.01). **(D)** Quantitative analysis of collagen fibers in Masson’s trichrome-stained sections (n = 6; ns, *P* > 0.05, **P < 0.01). **(E)** Protein expression of α-SMA and Collagen I in liver tissues of CCl_4_-induced fibrotic mice treated with AMD3100 and ALB. **(F)** Relative protein expression of α-SMA (n = 3; ns, *P* > 0.05, *P < 0.05). **(G)** Relative protein expression of Collagen I (n = 3; ns, *P* > 0.05, **P < 0.01). **(H)** Protein expression of CXCL12 and CXCR4 in liver tissues of CCl_4_-induced mice treated with AMD3100 and ALB. **(I)** Relative protein expression of CXCL12 (n = 3; ns, *P* > 0.05, *P < 0.05). **(J)** Relative protein expression of CXCR4 (n = 3; ns, *P* > 0.05, *P < 0.05). **(K–N)** Relative mRNA expression of inflammatory cytokines (n = 6; ns, *P* > 0.05, *P < 0.05, **P < 0.01).

Utilizing the potent and specific CXCR4 inhibitor AMD3100, we investigated the role of CXCL12/CXCR4 axis in the therapeutic effects of ALB on CCl_4_-induced mice (Wang et al., 2020). The liver fibrosis model and its corresponding therapeutic approaches are depicted in Figure 4A. Histopathological examinations demonstrated a reduction in hepatocyte necrosis, immune cell infiltration, and collagen deposition following treatments with ALB and AMD3100. Nevertheless, AMD3100 nullified the hepatoprotective effects observed with ALB alone (Figures 4B–D). Additionally, Western blotting assays revealed that AMD3100 diminished the protein expression of *α*-SMA and Collagen I (Figures 4E–G), and counteracted the inhibitory effects of ALB on the CXCL12/CXCR4 axis (Figures 4H–J). Similarly, AMD3100 reversed the suppression of the inflammasome in the liver by ALB (Figures 4K–N). These data indicated that the CXCR4 inhibitor AMD3100 can abrogate the hepatoprotective and anti-inflammatory effects of ALB, thereby providing evidence that ALB exerts its antifibrotic actions via the CXCL12/CXCR4 axis in CCl_4_ mice.

### ALB inhibited the inflammatory pathways in CCl_4_-induced mice

**FIGURE 5:**
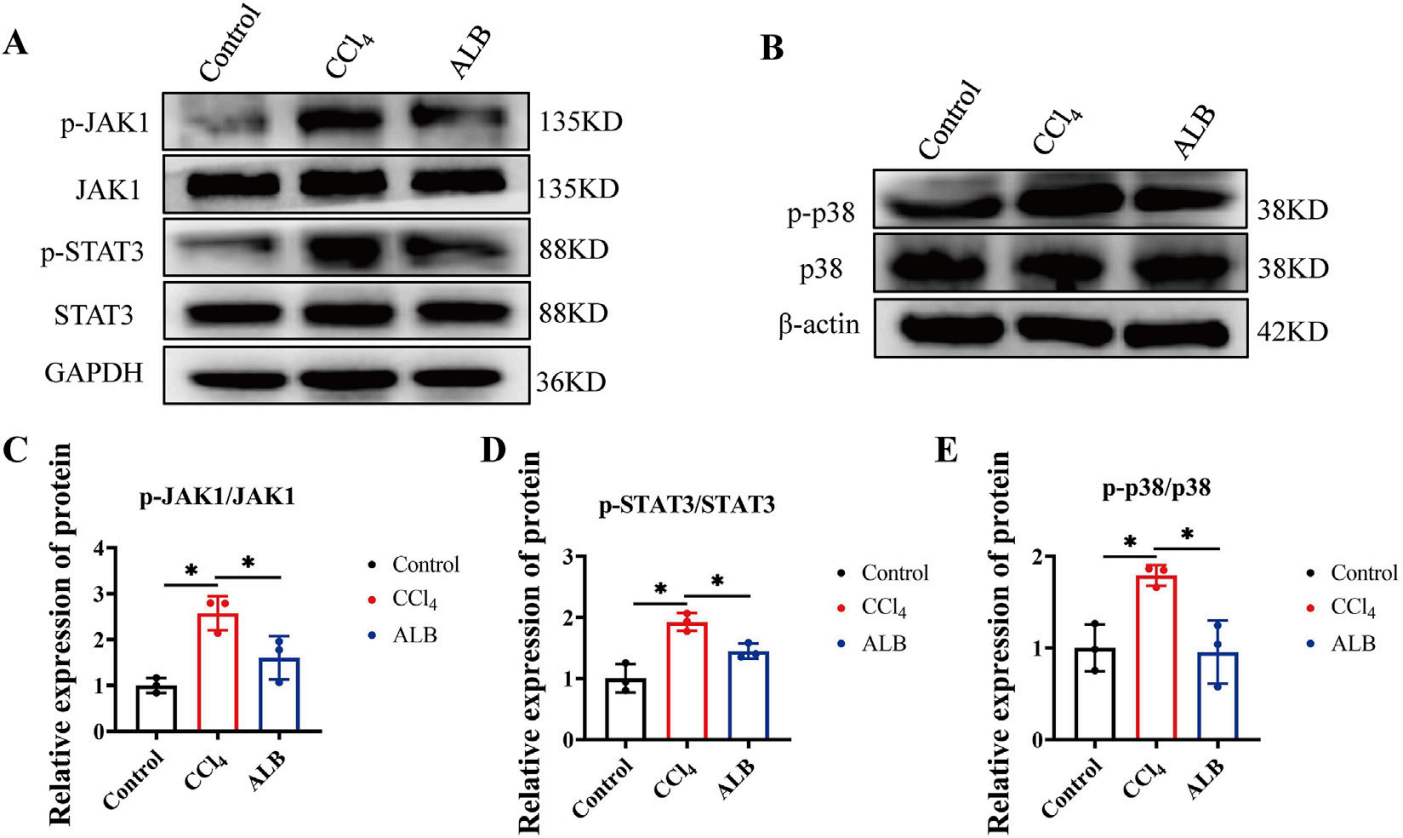
ALB inhibited the inflammatory pathways in CCl_4_-induced mice. **(A)** Protein expression of JAK1, p-JAK1, STAT3, and p-STAT3 in the livers of CCl_4_-induced mice treated with ALB. **(B)** Protein expression of p38 and p-p38 in the livers of CCl_4_-induced mice treated with ALB. **(C)** Relative protein expression of p-JAK1/JAK1 (n = 3; *P < 0.05). **(D)** Relative protein expression of p-STAT3/STAT3 (n = 3; *P < 0.05). **(E)** Relative protein expression of p-p38/p38 (n = 3; *P < 0.05).

The activation of JAK1/STAT3 and p38 MAPK pathways via the CXCL12/CXCR4 axis can further enhance the pro-inflammatory activity of liver cells (Cao et al., 2022; Ma et al., 2024). Therefore, we explored how ALB might protect the liver by regulating these pathways. Western blot assays showed ALB significantly reduced p-JAK1, p-STAT3, and p-p38 levels in CCl_4_-induced mice (Figures 5A–E). Indicating ALB’s anti-inflammatory and antifibrotic effects via the CXCL12/CXCR4-mediated pathways.

### The combination of ALB and MET synergistically inhibited the progression of liver fibrosis in mice via the CXCL12/CXCR4 axis

**FIGURE 6:**
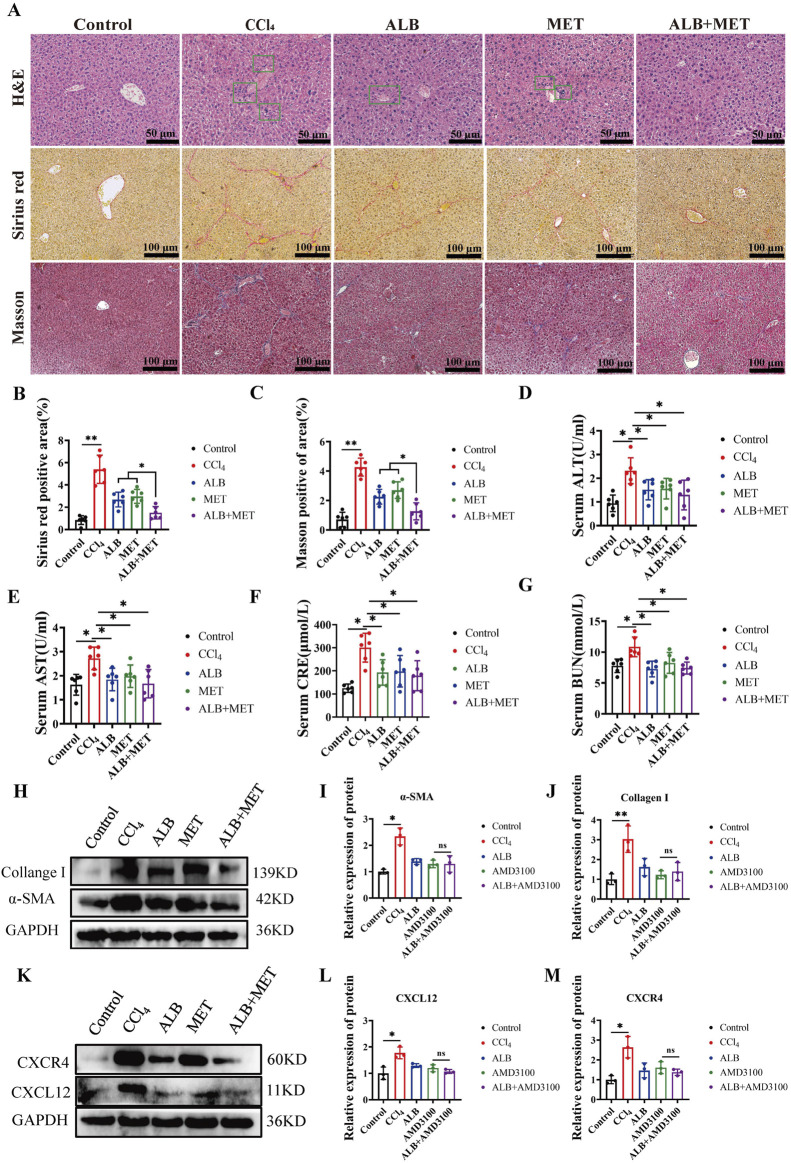
The combination of ALB and MET synergistically inhibited the progression of liver fibrosis in mice via the CXCL12/CXCR4 axis. **(A)** H&E staining (bar = 50 μm), Sirius Red staining (bar = 100 μm), and Masson’s trichrome staining (bar = 100 μm) of various experimental groups. **(B)** Quantitative analysis of collagen fibers in Sirius Red-stained sections (n = 6; ns, *P* > 0.05, **P < 0.01). **(C)** Quantitative analysis of collagen fibers in Masson’s trichrome-stained sections (n = 6; ns, *P* > 0.05, **P < 0.01). **(D)** Serum levels of ALT. **(E)** Serum levels of AST. **(F)** Serum levels of CRE. **(G)** Serum levels of BUN. **(H)** Protein expression of α-SMA and Collagen I in liver tissues of CCl_4_-induced mice treated with ALB and MET. **(I)** Relative protein expression of α-SMA (n = 3; ns, *P* > 0.05, *P < 0.05). **(J)** Relative protein expression of Collagen I (n = 3; ns, *P* > 0.05, **P < 0.01). **(K)** Protein expression of CXCL12 and CXCR4 in liver tissues of CCl_4_-induced mice treated with ALB and MET. **(L)** Relative protein expression of CXCL12 (n = 3; ns, *P* > 0.05, *P < 0.05). **(M)** Relative protein expression of CXCR4 (n = 3; ns, *P* > 0.05, *P < 0.05).

Given that ALB’s high aqueous solubility leads to diminished bioavailability and a delayed therapeutic effect, we endeavor to investigate potential strategies for its combined use with other drugs. MET, known primarily for its antidiabetic properties, also exhibits notable anti-fibrotic effects (Yang et al., 2023). Therefore, we evaluated the efficacy of ALB in combination with MET for treating liver fibrosis. he designated doses for this investigation are set at ALB (50 mg/kg) and MET (100 mg/kg). H&E staining results showed that the application of ALB or MET alone could restore the structure of damaged liver tissue; when both were used in combination, cell arrangement became more regular, and the liver tissue structure nearly returned to normal. Moreover, sirius red and masson trichrome staining showed that the combination of ALB and MET significantly decreased liver fibrosis compared to monotherapy (Figures 6A–C). Additionally, the serological study showed that the combined drug therapy did not have harmful effects on liver or kidney function. (Figures 6D–G). Western blot analysis further supported these findings, demonstrating that in the combined treatment group, the protein expression levels of α-SMA and Collagen I were significantly decreased, and lower than those in the respective monotherapy groups (Figures 6H–J). The research results on the mechanism of action indicated thatthe combination therapysignificantly downregulate the expression levels of key proteins CXCL12 and CXCR4 in a mouse model of liver fibrosis (Figures 6K–M). These findings suggested that the combination of ALB and MET may inhibit the progression of liver fibrosis through the suppression of the CXCL12/CXCR4 signaling pathway, thereby exerting a synergistic effect.

## Discussion

Liver fibrosis, as a critical pathological manifestation of chronic liver disease, involves complex cellular and molecular mechanisms and is one of the key factors contributing to increased liver-related morbidity and mortality (Li S. et al., 2024). Given the current lack of highly effective, specific treatments for liver fibrosis in the medical field, exploring effective adjunctive therapeutic strategies is particularly urgent (Jiang et al., 2023). Owing to the structural diversity and broad spectrum of biological activities, as well as their favorable biocompatibility, natural products demonstrate considerable advantages in the treatment of diseases such as liver fibrosis (Zhang et al., 2024). ALBis a monoterpene glycoside predominantly found in PRR, characterized by its unique cage-like pinane skeleton structure, which exhibits marked anti-inflammatory activity (Zhang and Wei, 2020). In this study, we found that ALB effectively mitigates liver fibrosis by modulating the CXCL12/CXCR4 axis, thereby inhibiting the expression of profibrotic markers and reducing inflammatory responses. Additionally, the combination of ALB with MET was found to further suppress the progression of liver fibrosis. These results suggested that ALB has significant potential as a therapeutic candidate for liver diseases.

CCl_4_is a widely utilized modeling agent for inducing liver fibrosis. Its metabolic activation generates free radicals that trigger lipid peroxidation in the liver, leading to hepatocellular injury and subsequent fibrogenesis (Xu et al., 2024). Our study showed that ALB significantly reduced CCl_4_-induced increases serum ALT, AST, CRE and BUN levels and mitigated liver damage, as confirmed by H&E staining. We also found that ALB treatment significantly decreased collagen deposition and abnormal collagen fiber proliferation in the liver compared to CCl_4_mice.The activation of HSCs is a primary pathogenic mechanism in liver fibrosis (Liepelt and Tacke, 2016). Continuous liver injury caused by CCl_4_ leads to the release of pro-inflammatory factors, which in turn trigger HSC activation and ECM accumulation, ultimately resulting in liver fibrosis. Our results demonstrated that ALBreduced α-SMA and Collagen I protein expression in CCl_4_-treated mice and inhibited TGF-β induced activation of LX-2 cells in a dose-dependent manner. These results suggested that ALB has significant potential as a therapeutic candidate for liver diseases.

Transcriptome sequencing and RT-qPCR analysis showed that the CXCL12/CXCR4 axis experienced the most notable changes in the liver of CCl_4_-induced mice after ALB treatment. Inflammatory chemokines, such as CXCL12, play a key role in liver disease by regulating immune responses and influencing fibrosis (Hammerich and Tacke, 2023). CXCL12, also known as SDF-1, interacts with its primary receptor CXCR4, which is mainly found on fibroblasts. Both are involved in various physiological and pathological processes (Zhang W. S. et al., 2022). Currently, there is evidence demonstrating that natural products exert anti-fibrotic effects by targeting the CXCL12/CXCR4 axis (Qin et al., 2018). In this study, we found that ALB significantly downregulates CXCL12 and CXCR4 protein expression and results in a marked reduction of pro-inflammatory cytokines, including IL-6, IL-1β, TNF-α, and NLRP3. These results suggest that ALB mitigates inflammation and ameliorates liver fibrosis by targeting the CXCL12/CXCR4 axis, a finding confirmed by experiments using the CXCR4 inhibitor AMD3100 in CCl_4_-treated mice. Studies have shown that ALB alleviates DSS-induced ulcerative colitis in mice by reducing inflammation and oxidative stress through the p38 MAPK pathway (Wang et al., 2022). Additionally, TPG (Total Glycoside of Paeony), with ALB as its main component, has anti-inflammatory effects via the JAK2/STAT3 pathway (Zhang and Wei, 2020). Both pathways are linked to the CXCL12/CXCR4 axis. Our study found ALB reverses increased p-STAT3 and p-p38 levels in CCl_4_-induced fibrotic mice, indicating its antifibrotic effects through the STAT3 and p38 MAPK pathways.

The combination therapy regimen has garnered significant attention in recent years as an innovative strategy for the treatment of liver fibrosis. For instance, the combined use of the entecavir with the Biejia-Ruangan compound has been demonstrated to significantly improve liver fibrosis in patients with chronic hepatitis B (Ji et al., 2022). MET not only demonstrates significant hypoglycemic effects but also exhibits multiple biological activities, including anti-inflammatory (Petrasca et al., 2023), lifespan-extension (Xiao et al., 2022), and enhancing innate immunity (Xiao et al., 2020) properties. In combination therapy, the triad of MET, the insulin sensitizer pioglitazone, and the GLP-1 receptor agonist exenatide has achieved positive outcomes in reducing the incidence of liver fibrosis and steatosis (Lavynenko et al., 2022). Building on this, we explored the effects of low-dose ALB and MET on CCl_4_-induced liver fibrosis (using dosages of 50 mg/kg for ALB and 100 mg/kg for MET). Measuring serum ALT, AST, CRE, and BUN levels helps evaluate liver and kidney function. Our study found that CCl_4_-treated mice had increased enzyme levels, indicating liver damage. However, treatment with ALB, MET, and their combination significantly reduced these enzyme levels. Furthermore, Histopathological and Western blot analyses showed that the combination of ALB and MET was more effective against liver fibrosis than either drug alone. These results indicated that an ALB-supplemented regimen alleviates liver fibrosis in CCl_4_-induced mice, highlighting its potential as an adjuvant therapy.

**FIGURE 7:**
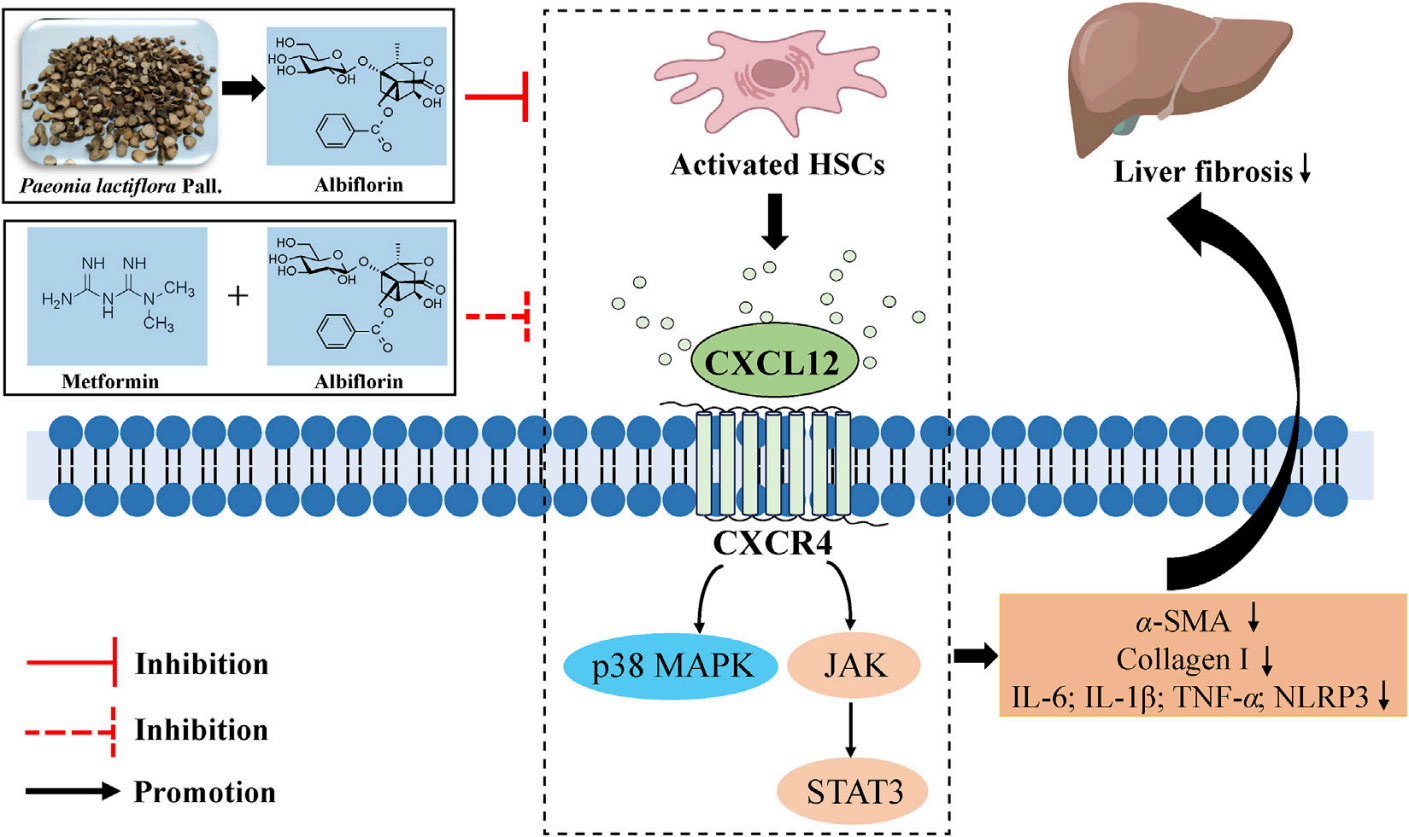
Schematic Diagram of the antifibrotic effect of ALB and its Combination with MET.

In conclusion, our study reveals that ALB inhibits inflammation and improves liver fibrosis by targeting the CXCL12/CXCR4 axis in CCl_4_-induced mice. Additionally, the combination of ALB and metformin synergistically inhibits the progression of liver fibrosis (Figure 7). Our findings further indicate the potential clinical application of ALB as an antifibrotic drug.

## Data Availability

The datasets presented in this study can be found in online repositories. The names of the repository/repositories and accession number(s) can be found in the article/Supplementary Material.
