# Supplementary material for: Albiflorin inhibits inflammation to improve liver fibrosis by targeting the CXCL12/CXCR4 axis in mice
Source: Front Pharmacol. 2025 Apr 30;16:1577201. doi: 10.3389/fphar.2025.1577201 (PMC12074940; doi:10.3389/fphar.2025.1577201)
Supplement: Supplementary file 1 [file DataSheet2.pdf]

Figure 1I

Original western bolt bands

Control Model ALB paeoniflorin

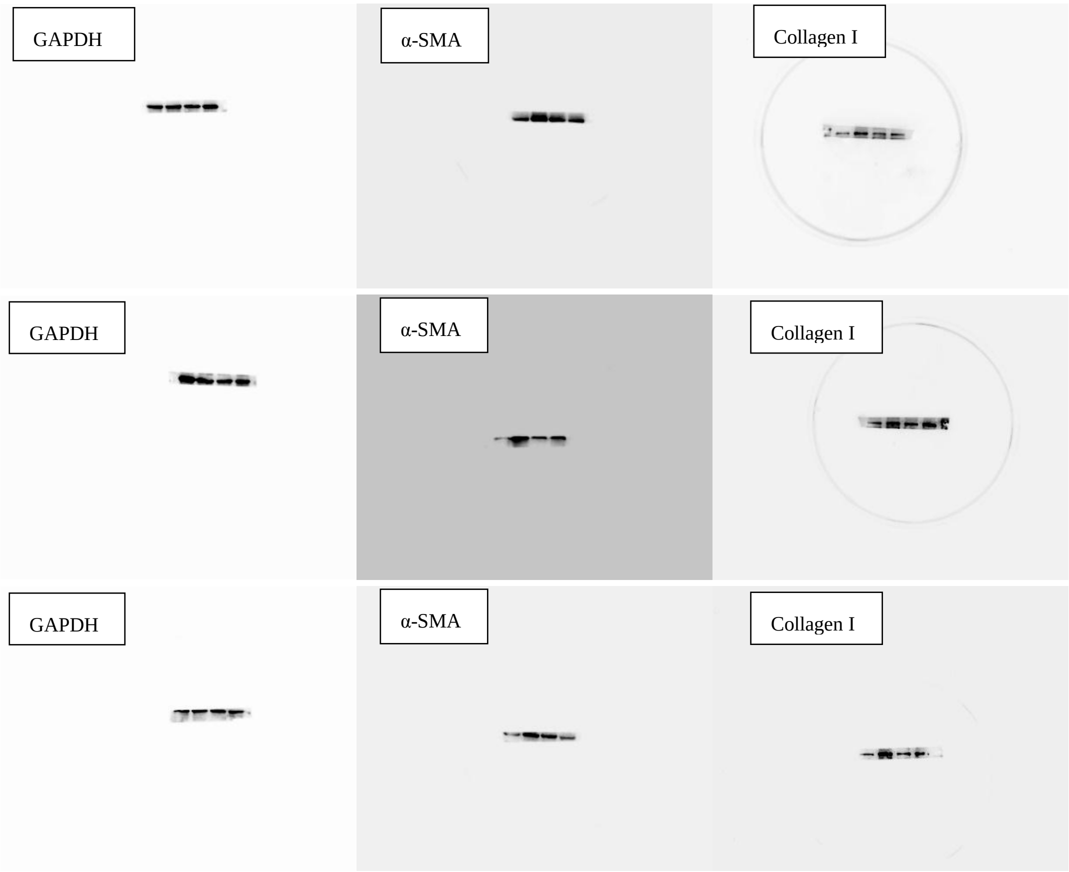

Figure 2A and 2G

Original western bolt bands

ALB(μM)	-	-	12.5	25	50
TGF-β1	-	+	+	+	+

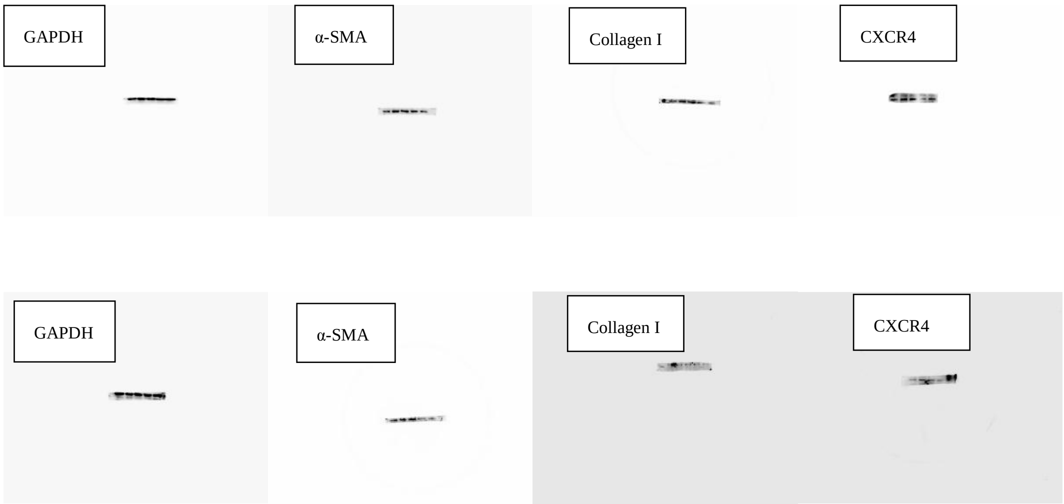

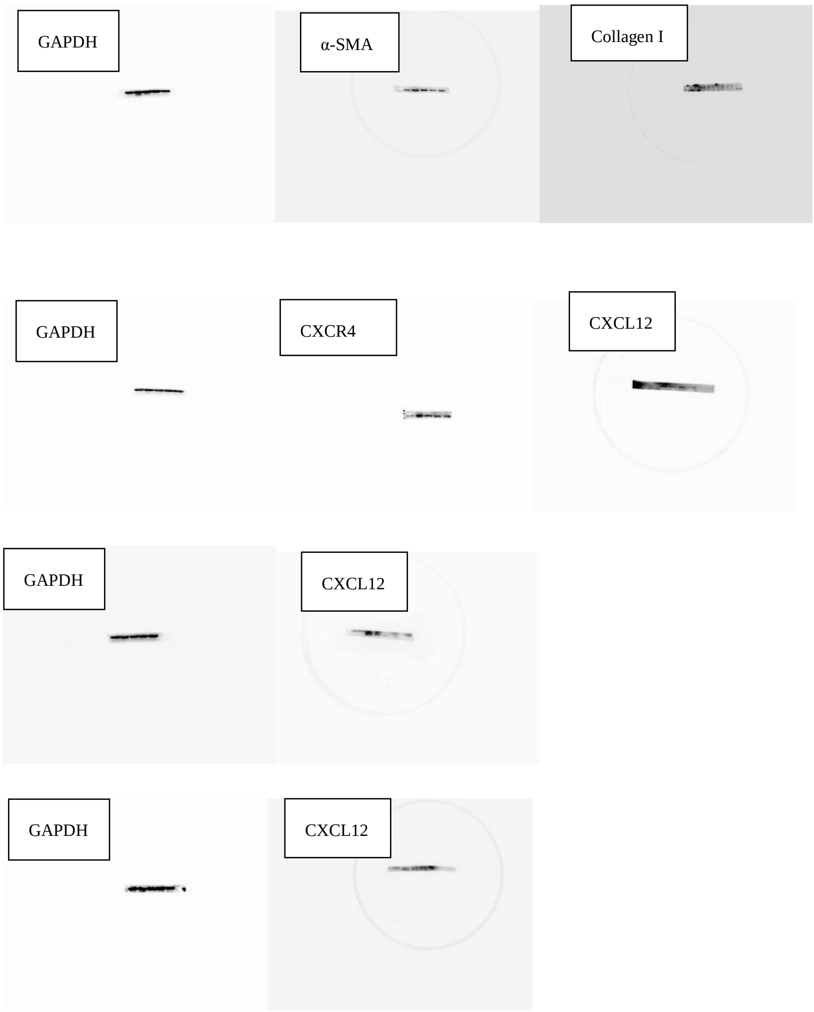

Figure 3A

Original western bolt bands

Control Model ALB

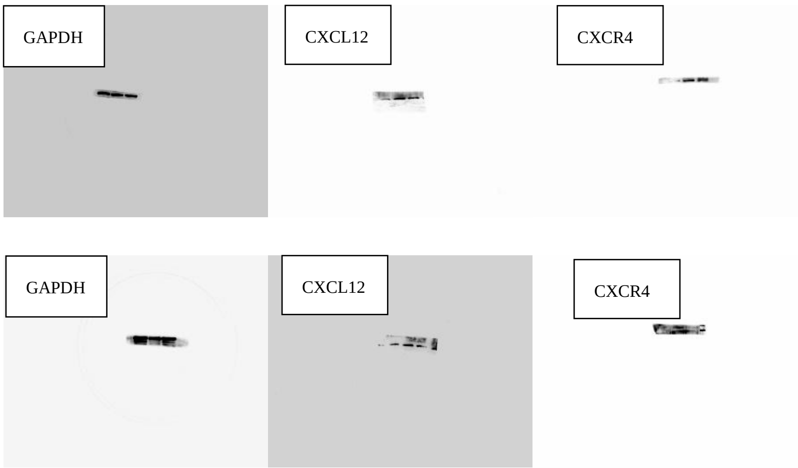

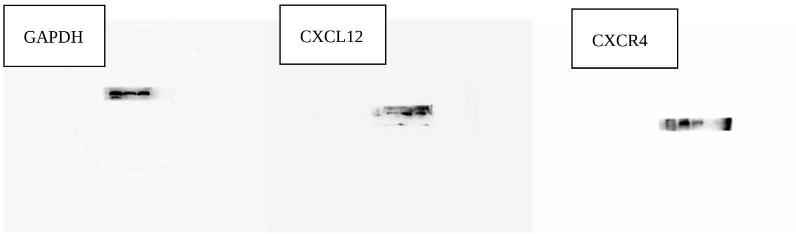

Figure 4E and H

Original western bolt bands

Control Model ALB AMD3100 ALB+AMD3100

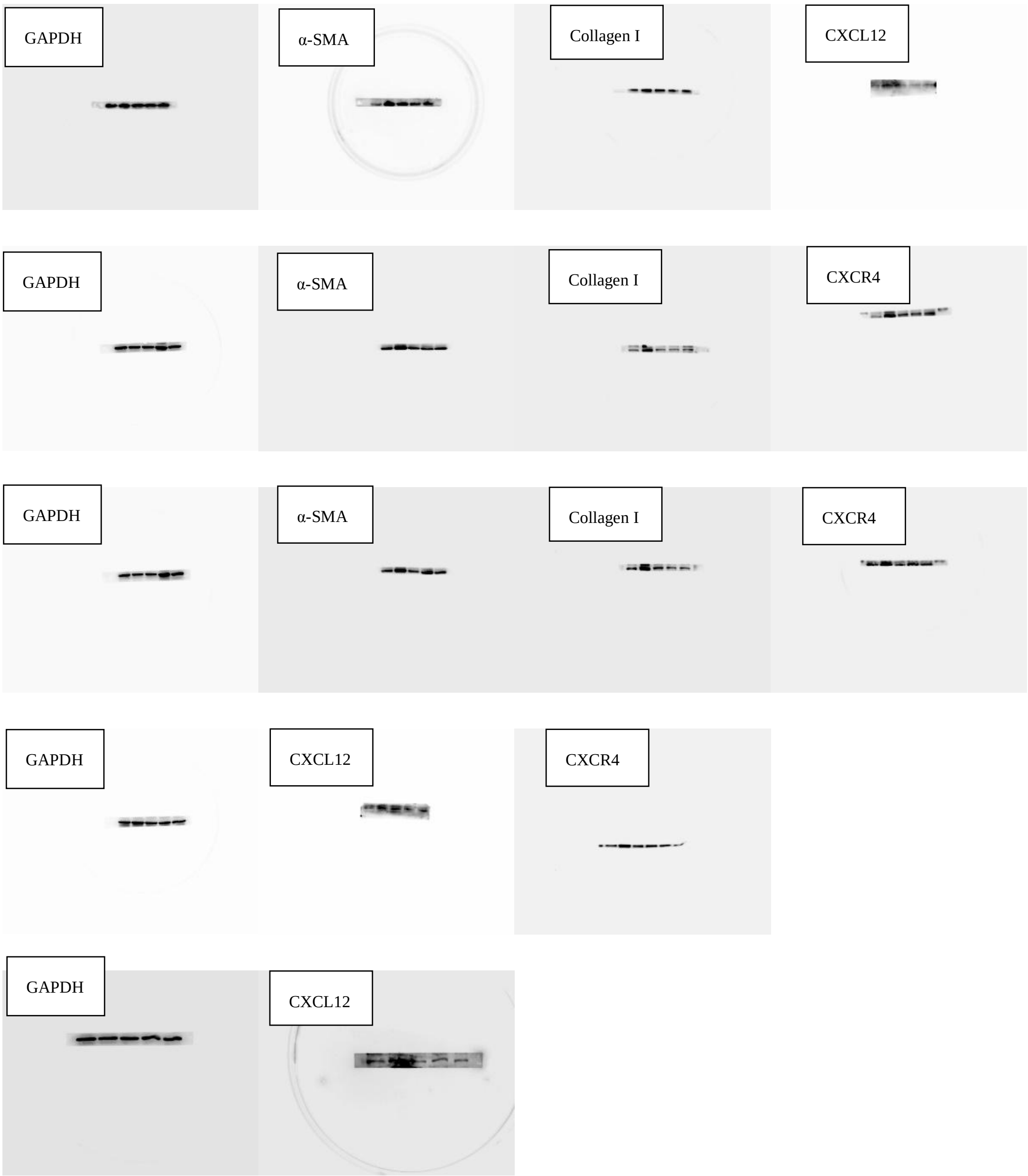

Figure 5A

Original western bolt bands

Control Model ALB

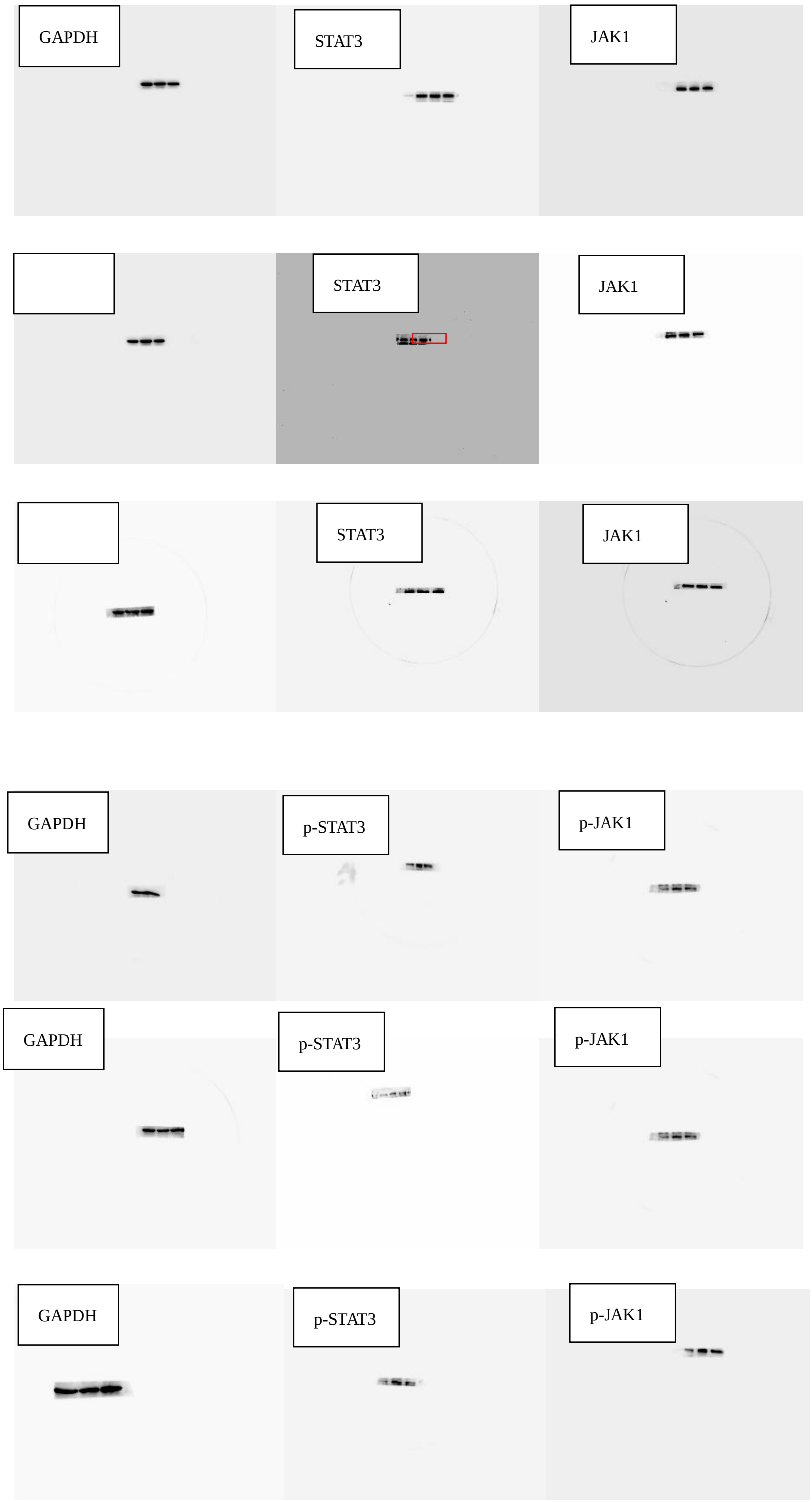

Figure5B

Original western bolt bands

Control   Model ALB

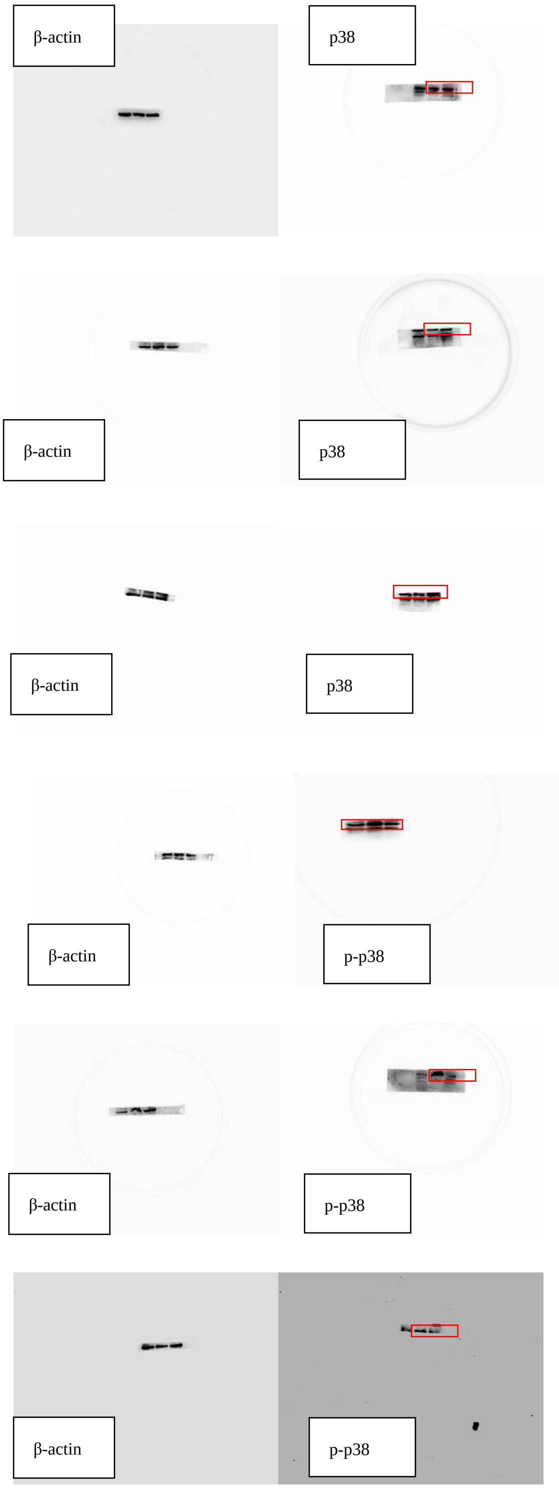

Figure 6H

Original western bolt bands

Control   Model ALB   MET   ALB+MET

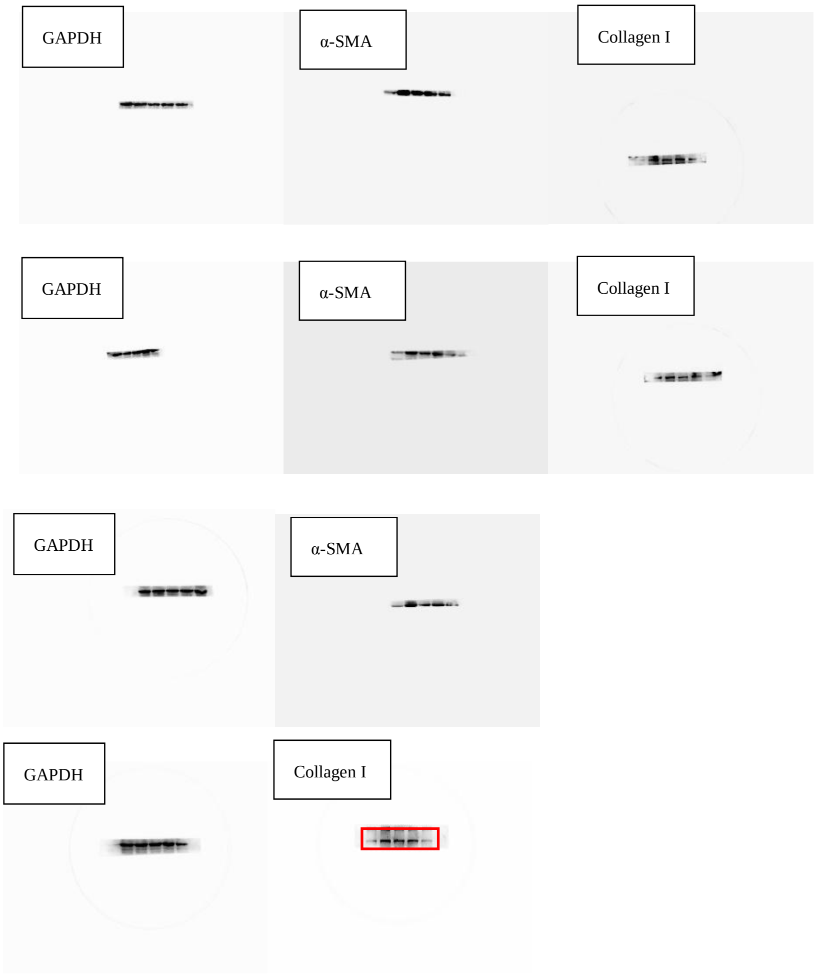

Figure 6K

Original western bolt bands

Control   Model ALB   MET   ALB+MET

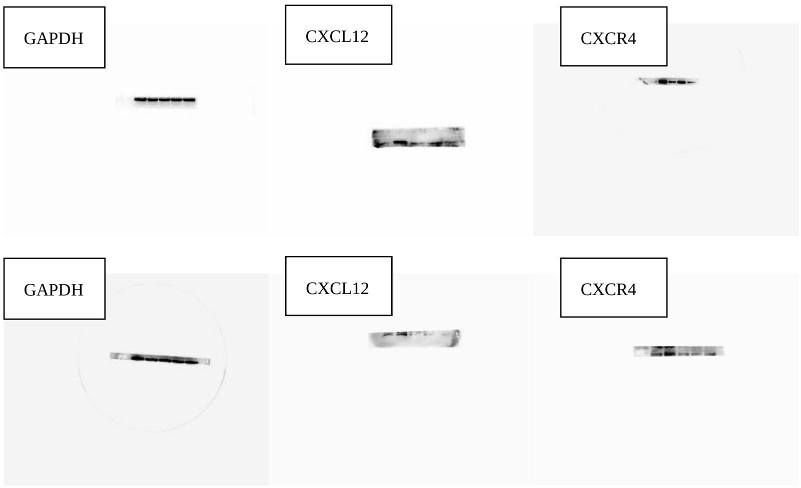

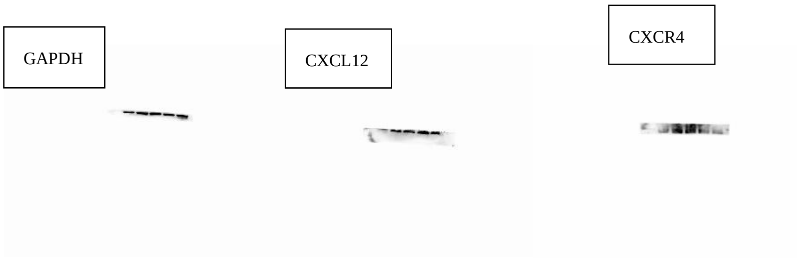
GAPDH
CXCL12
CXCR4
