# Supplementary material for: Albiflorin inhibits inflammation to improve liver fibrosis by targeting the CXCL12/CXCR4 axis in mice
Source: Front Pharmacol. 2025 Apr 30;16:1577201. doi: 10.3389/fphar.2025.1577201 (PMC12074940; doi:10.3389/fphar.2025.1577201)
Supplement: Supplementary file 2 [file Table1.docx]

| Gene | Primer sequences (5′→3′) | |
| --- | --- | --- |
| *Il-1β* | Forward | CCCTGCAGCTGGAGAGTGTGGA |
|  | Reverse | TGTGCTCTGCTTGTGAGGTGCTG |
| *Il-6* | Forward | CGGAGAGGAGACTTCACAGAGGA |
|  | Reverse | TTTCCACGATTTCCCAGAGAACA |
| *TNF-α* | Forward | TGACAAGCCTGTAGCCCACG |
|  | Reverse | TTGTCTTTGAGATCCATGCCG |
| *Nlrp3* | Forward | ATCAACAGGCGAGACCTCTG |
|  | Reverse | GTCCTCCTGGCATACCATAGA |
| *Cxcl12* | Forward | TGCATCAGTGACGGTAAACCA |
|  | Reverse | CACAGTTTGGAGTGTTGAGGAT |
| *Cxcr4* | Forward | ATTGTCCACGCCACCAACAG |
|  | Reverse | ACATCGGCGAAGATGATGTCAG |
| *Gapdh* | Forward | TTGATGGCAACAATCTCCAC |
|  | Reverse | CGTCCCGTAGACAAAATGGT |
| *CCL2* | Forward | CAGCCAGATGCAATCAATGCC |
|  | Reverse | TGGAATCCTGAACCCACTTCT |
| *CCL3* | Forward | AGTTCTCTGCATCACTTGCTG |
|  | Reverse | CGGCTTCGCTTGGTTAGGAA |
| *CCL3L1* | Forward | CACCTCCCGACAGATTCCAC |
|  | Reverse | GGTCACTGACGTATTTCTGGAC |
| *CCR10* | Forward | GCTTTGCTACAAGGCCGATG |
|  | Reverse | GGAGACACTGGGTTGGAAGG |
| *CXCL10* | Forward | GTGGCATTCAAGGAGTACCTC |
|  | Reverse | TGATGGCCTTCGATTCTGGATT |
| *CXCL11* | Forward | GACGCTGTCTTTGCATAGGC |
|  | Reverse | GGATTTAGGCATCGTTGTCCTTT |
| *CXCL12* | Forward | ATTCTCAACACTCCAAACTGTGC |
|  | Reverse | ACTTTAGCTTCGGGTCAATGC |
| *GAPDH* | Forward | AATGAAGGGGTCATTGATGG |
|  | Reverse | AAGGTGAAGGTCGGAGTCAA |

**Table S1** Primer Sequences for q-PCR
