# Supplementary material for: Albiflorin inhibits inflammation to improve liver fibrosis by targeting the CXCL12/CXCR4 axis in mice
Source: Front Pharmacol. 2025 Apr 30;16:1577201. doi: 10.3389/fphar.2025.1577201 (PMC12074940; doi:10.3389/fphar.2025.1577201)

**Figure S1 RNA-seq transcriptome analysis of ALB.**

(A) Volcano map showing the distribution of different genes in the ALB and model groups. The upregulated genes are represented by red dots, and the downregulated genes are represented by green dots. (B) Differential gene cluster analysis (heatmap); the redder the color, the higher the expression; the bluer the color, the lower the expression.


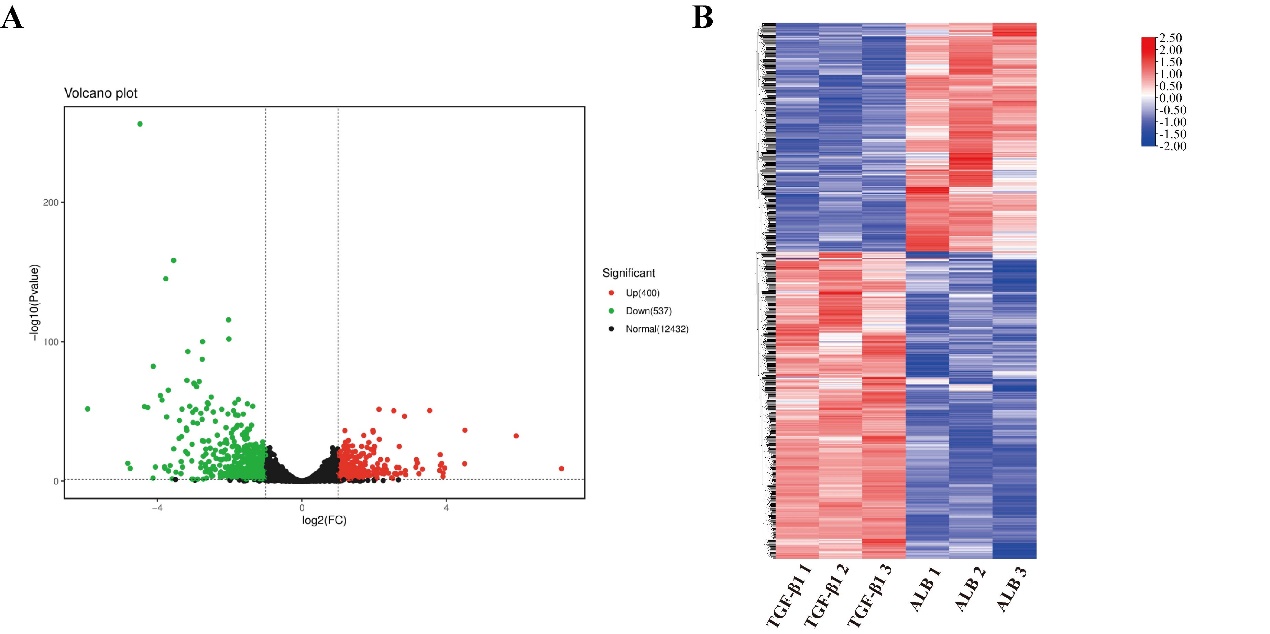


**Figure S2 Effect of ALB on proliferation of hepatic stellate cells LX-2.**

"*" indicates that compared with the not dosing group, **P* < 0.05, ns > 0.05, n = 3.


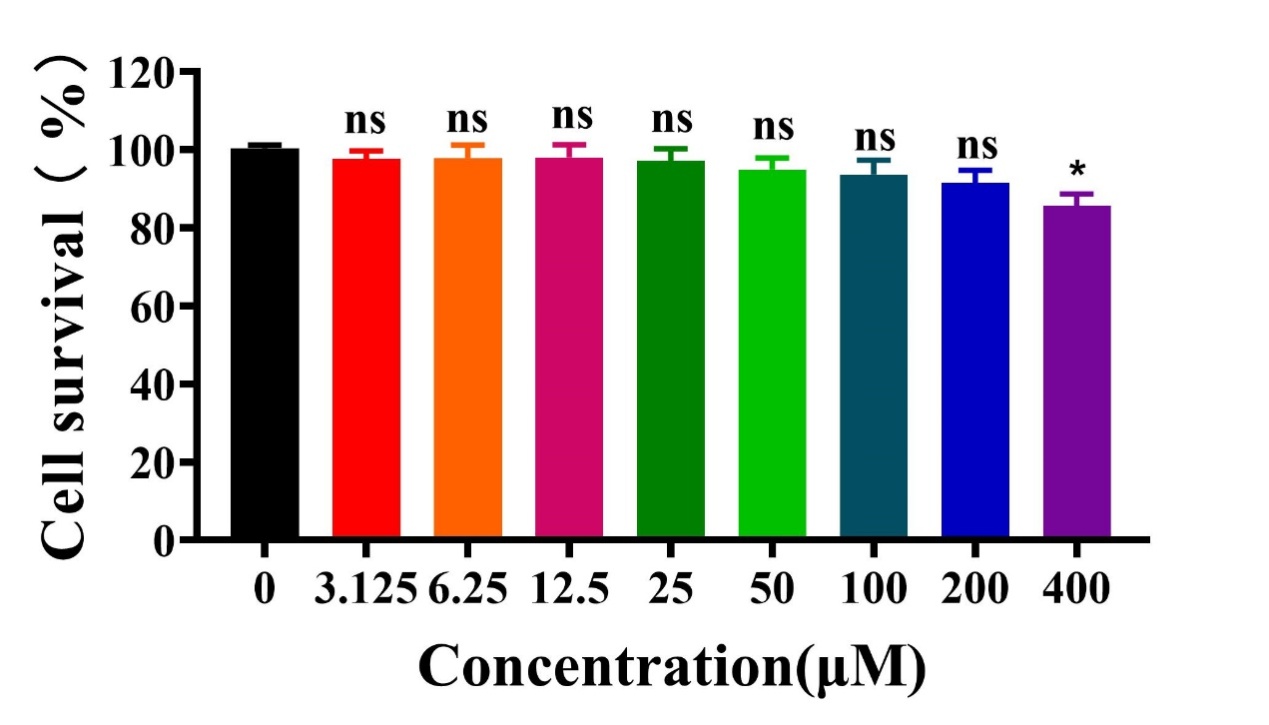

Supplement: Supplementary file 3 [file DataSheet1.docx]
